# Changes in Management Strategy and Impact of Neoadjuvant Therapy on Extent of Surgery in Invasive Lobular Carcinoma of the Breast: Analysis of the National Cancer Database (NCDB)

**DOI:** 10.1245/s10434-021-09715-3

**Published:** 2021-03-09

**Authors:** Rita A. Mukhtar, Tanya L. Hoskin, Elizabeth B. Habermann, Courtney N. Day, Judy C. Boughey

**Affiliations:** 1 Department of Surgery, University of California, San Francisco, CA USA; 2 Department of Health Sciences Research, Mayo Clinic, Rochester, MN USA; 3 Department of Surgery, Mayo Clinic, Rochester, MN USA; 4 Robert D. and Patricia E. Kern Center for the Science of Health Care Delivery, Mayo Clinic, Rochester, MN USA

## Abstract

**Background:**

Given reports of low response rates to neoadjuvant chemotherapy (NAC) in invasive lobular carcinoma (ILC), we evaluated whether use of alternative strategies such as neoadjuvant endocrine therapy (NET) is increasing. Additionally, we investigated whether NET is associated with more breast conservation surgery (BCS) and less extensive axillary surgery in those with ILC.

**Patients and Methods:**

We queried the NCDB from 2010 to 2016 and identified all women with stage I–III hormone receptor positive, human epidermal growth factor receptor-2 negative (HR+/HER2−) ILC who underwent surgery. We used Cochrane–Armitage tests to evaluate trends in utilization of the following treatment strategies: NAC, short-course NET, long-course NET, and primary surgery. We compared rates of BCS and extent of axillary surgery stratified by clinical stage and tumor receptor subtype for each treatment strategy.

**Results:**

Among 69,312 cases of HR+/HER2− ILC, NAC use decreased slightly (from 4.7 to 4.2%, *p* = 0.007), while there was a small but significant increase in long-course NET (from 1.6 to 2.7%, *p* < 0.001). Long-course NET was significantly associated with increased BCS in patients with cT2–cT4 disease and less extensive axillary surgery in clinically node positive patients with HR+/HER2− tumors.

**Conclusions:**

Primary surgery remains the most common treatment strategy in patients with ILC. However, NAC use decreased slightly over the study period, while the use of long-course NET had a small increase and was associated with more BCS and less extensive axillary surgery.

Use of systemic therapy prior to surgical resection can improve outcomes and provide prognostic information for many women with breast cancer. Neoadjuvant chemotherapy (NAC) increases breast conservation rates, and achieving a pathologic complete response (pCR) after NAC is associated with improved disease-free survival. In recent years, a newer approach of using neoadjuvant endocrine therapy (NET) has emerged as a treatment strategy for hormone receptor (HR)-positive tumors.^1^ When given for 3–6 months, NET can potentially downstage the tumor in the breast and/or axilla, affording the opportunity to decrease the extent of surgical intervention.^2^

When short courses of NET (2–4 weeks) are used, investigators have shown that changes in biomarkers such as the proliferation marker Ki67 can predict improved long-term outcomes and potentially guide adjuvant therapy decisions.^3^,^4^ The POETIC trial (perioperative endocrine therapy—individualizing care) is evaluating the relationship between change in Ki67 after 2 weeks of endocrine therapy and long-term outcomes in over 4000 post-menopausal women with HR-positive breast cancer with the goal of identifying patients who would benefit from additional adjuvant therapy.^5^–^7^

Of these strategies, selecting the optimal treatment approach (surgery first, NAC, short-course, or long-course NET) for an individual patient, especially those with HR+ disease, remains a clinical challenge.^8^,^9^ For women with invasive lobular carcinoma (ILC) in particular, limited data exist to guide optimal management.^10^ ILC is the second most common subtype of breast cancer, representing approximately 10–15% of all breast cancers. ILC has unique features, and is characterized by its diffuse growth pattern in so called “single file lines,” resulting from the absence of the adhesion protein E-cadherin.^11^ It differs from the more common invasive ductal carcinoma in its mutational profile, appearance on imaging, surgical outcomes, and timing/pattern of recurrence.^11^–^14^ Many studies have shown poor responses to NAC in ILC with low pCR rates.^15^–^17^ Given that most ILC are estrogen receptor (ER)-positive and human epidermal growth factor receptor-2 (HER2)-negative tumors, the poor overall response to NAC has led to interest in the use of NET in this tumor type.^18^ But, whether this approach is being utilized (either in the short or long durations described) in ILC is unknown. We sought to evaluate the primary management strategies used for ILC, including duration of NET, and how these have changed over time. Additionally, we investigated which surgical procedures were associated with each management strategy.

## Patients and Methods

### Study Design

The National Cancer Database (NCDB) is a deidentified national database sponsored by the American College of Surgeons and the American Cancer Society, containing clinicopathologic data and outcomes collected from 1500 accredited cancer centers. The data therein represent approximately 70% of newly diagnosed cancer cases in the USA.

**Fig. 1:**
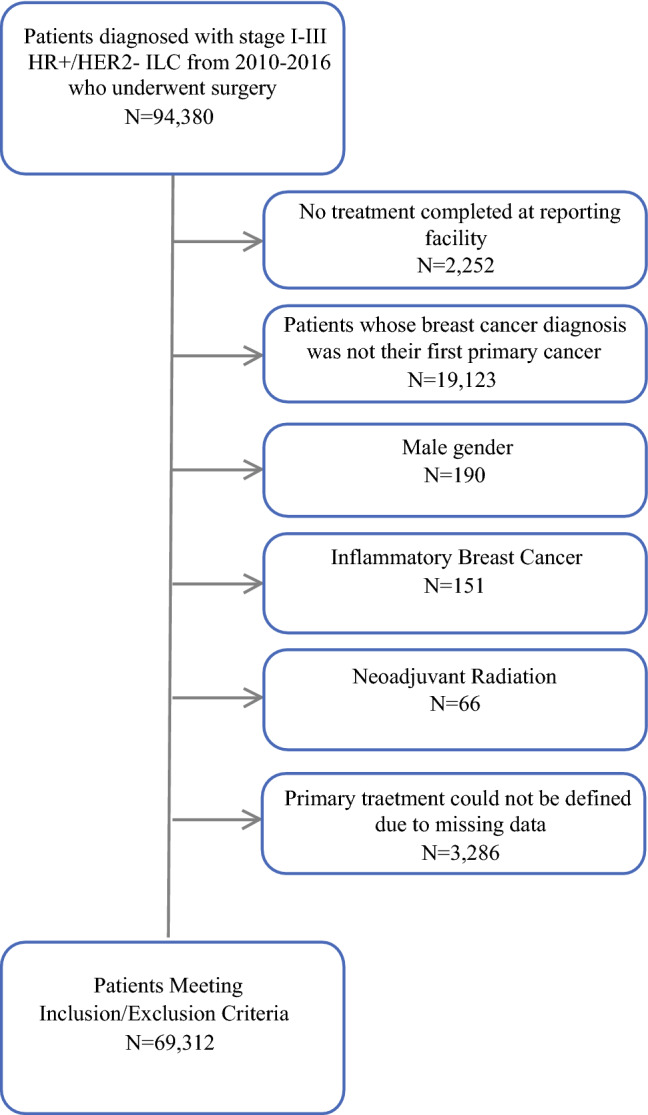
Flowchart showing cohort selection

With institutional review board exemption provided by both participating institutions, we queried the NCDB (2016 PUF) from 2010 to 2016 and identified all patients with clinical stage I–III HR+/HER2− ILC who underwent surgery. Patients with HER2+ and triple-negative ILC were excluded from our primary analysis sample since they make up a small minority of all ILC cases, but these patients were described separately. Cases with prior cancer history (breast or otherwise), those receiving no treatment at the reporting facility, those with male gender, those with mixed invasive ductal/lobular histology, and those under the age of 18 years were excluded. Primary treatment strategy was classified as primary surgery, NAC, short-course NET, or long-course NET by comparing the timing of surgery relative to chemotherapy and hormone therapy start times. Cases were categorized as undergoing primary surgery if they had no systemic treatment prior to surgery. Patients who received chemotherapy starting 31–365 days before surgery constituted the NAC cohort, those who received endocrine therapy for 7–30 days before surgery constituted the short-course NET cohort, and those who received endocrine therapy for 31–365 days before surgery constituted the long-course NET cohort. Patients for whom primary treatment strategy could not be fully defined due to missing data regarding timing were also excluded (Fig. 1). Within the long-course NET cohort, we analyzed the association between the following treatment duration groups and surgical therapy received based on prior reports studying NET duration: 1–3 months, 3–6 months, 6–9 months, and > 9 months.^19^

ER and progesterone receptor (PR) immunohistochemical staining of ≥ 1% was classified as positive. Tumors that were ER+ and/or PR+ were classified as hormone receptor positive; tumors that were both ER− and PR− were classified as HR negative.

Our primary study questions were (1) whether the primary treatment strategy for ILC changed over time, (2) whether a particular strategy was associated with higher rates of breast conservation surgery (BCS), and (3) association of management strategy with extent of axillary surgery.

### Statistical Analysis

Cochrane–Armitage tests were used to assess treatment trends across time. Variables associated with primary treatment strategy were assessed in multivariable analysis using logistic regression. The relationship of primary treatment strategy with breast operation (BCS versus mastectomy) was assessed by clinical T category using chi-square tests for univariate analysis and with logistic regression models including a treatment by clinical T category interaction for multivariable analysis. Impact of primary treatment strategy on extent of axillary surgery (1–5 vs. > 5 nodes removed) was assessed similarly using clinical N status (positive or negative) as a stratification factor. Additional variables included in the multivariable model were year of diagnosis, age (years), tumor grade, tumor multicentricity, Charlson–Deyo comorbidity score, race, ethnicity, and insurance status. We included all standard clinicopathologic variables with significant association with treatment strategy on univariate analysis in the multivariable model. Finally, we conducted exploratory analyses on the small subset of patients with triple-negative (ER negative, PR negative, HER2 negative) or HER2-overexpressing ILC. Results are reported as odds ratios (OR) with 95% confidence intervals (CI). Analysis was performed using SAS (Version 9.4, SAS Institute Inc., Cary, NC). *p*-values < 0.05 were considered statistically significant.

## Results

### Patient and Tumor Characteristics

Of the 69,312 cases of HR+/HER2− ILC from 2010 to 2016, the average age was 63.1 years (range 22–90 years). Most patients had clinical stage I disease (60.5%). Additional patient and tumor characteristics are presented in Table 1.

**Table 1:** Patient and tumor characteristics of study cohort

	Total (*N* = 69,312)	Primary surgery (*N* = 64,460)	NAC (*N* = 3146)	NHT short (*N* = 305)	NHT long (*N* = 1401)
Age at diagnosis (years)					
*N*	69,312	64,460	3146	305	1401
Mean (SD)	63.0 (11.9)	63.3 (11.9)	56.0 (10.5)	65.1 (12.3)	67.2 (11.5)
Median	63.0	64.0	56.0	65.0	67.0
Q1, Q3	54.0, 72.0	54.0, 72.0	48.0, 64.0	56.0, 73.0	59.0, 76.0
Range	(22.0–90.0)	(22.0–90.0)	(24.0–90.0)	(34.0–90.0)	(35.0–90.0)
Spanish/Hispanic origin					
Missing	1972	1850	76	10	36
Not Spanish/Hispanic	64,309 (95.5%)	59,905 (95.7%)	2839 (92.5%)	277 (93.9%)	1288 (94.4%)
Spanish/Hispanic	3031 (4.5%)	2705 (4.3%)	231 (7.5%)	18 (6.1%)	77 (5.6%)
Race					
Missing	529	481	34	3	11
White	60,524 (88.0%)	56,411 (88.2%)	2628 (84.4%)	273 (90.4%)	1212 (87.2%)
Black	6077 (8.8%)	5566 (8.7%)	365 (11.7%)	21 (7.0%)	125 (9.0%)
Other	2182 (3.2%)	2002 (3.1%)	119 (3.8%)	8 (2.6%)	53 (3.8%)
Charlson–Deyo score					
0	57,836 (83.4%)	53,717 (83.3%)	2737 (87.0%)	240 (78.7%)	1142 (81.5%)
1	9219 (13.3%)	8637 (13.4%)	346 (11.0%)	48 (15.7%)	188 (13.4%)
2+	2257 (3.3%)	2106 (3.3%)	63 (2.0%)	17 (5.6%)	71 (5.1%)
Primary payer					
Missing	680	616	46	1	17
Not insured	871 (1.3%)	748 (1.2%)	90 (2.9%)	3 (1.0%)	30 (2.2%)
Private insurance/managed care	34,921 (50.9%)	32,293 (50.6%)	1980 (63.9%)	139 (45.7%)	509 (36.8%)
Medicaid	3229 (4.7%)	2854 (4.5%)	287 (9.3%)	14 (4.6%)	74 (5.3%)
Medicare	28,932 (42.2%)	27,342 (42.8%)	686 (22.1%)	144 (47.4%)	760 (54.9%)
Other government	679 (1.0%)	607 (1.0%)	57 (1.8%)	4 (1.3%)	11 (0.8%)
Clinical T category					
Missing	5309	5124	130	18	37
cT1	38,698 (60.5%)	38,003 (64.0%)	288 (9.5%)	122 (42.5%)	285 (20.9%)
cT2	18,435 (28.8%)	16,660 (28.1%)	1063 (35.2%)	101 (35.2%)	611 (44.8%)
cT3/4	6870 (10.7%)	4673 (7.9%)	1665 (55.2%)	64 (22.3%)	468 (34.3%)
Clinical node status					
Missing	4134	3949	128	14	43
cN0	56,955 (87.4%)	54,181 (89.5%)	1434 (47.5%)	243 (83.5%)	1097 (80.8%)
cN+	8223 (12.6%)	6330 (10.5%)	1584 (52.5%)	48 (16.5%)	261 (19.2%)
Grade					
Missing	5578	5004	423	25	126
Well differentiated	18,244 (28.6%)	17,051 (28.7%)	665 (24.4%)	89 (31.8%)	439 (34.4%)
Moderately differentiated	41,253 (64.7%)	38,545 (64.8%)	1762 (64.7%)	180 (64.3%)	766 (60.1%)
Poorly differentiated/undifferentiated	4237 (6.6%)	3860 (6.5%)	296 (10.9%)	11 (3.9%)	70 (5.5%)
ER status					
Positive	69,244 (99.9%)	64,402 (99.9%)	3137 (99.7%)	304 (99.7%)	1401 (100.0%)
Negative	68 (0.1%)	58 (0.1%)	9 (0.3%)	1 (0.3%)	0 (0.0%)
PR status					
Positive	60,509 (87.3%)	56,356 (87.4%)	2709 (86.1%)	265 (86.9%)	1179 (84.2%)
Negative	8803 (12.7%)	8104 (12.6%)	437 (13.9%)	40 (13.1%)	222 (15.8%)

### Trends in Primary Treatment Strategy

**Fig. 2:**
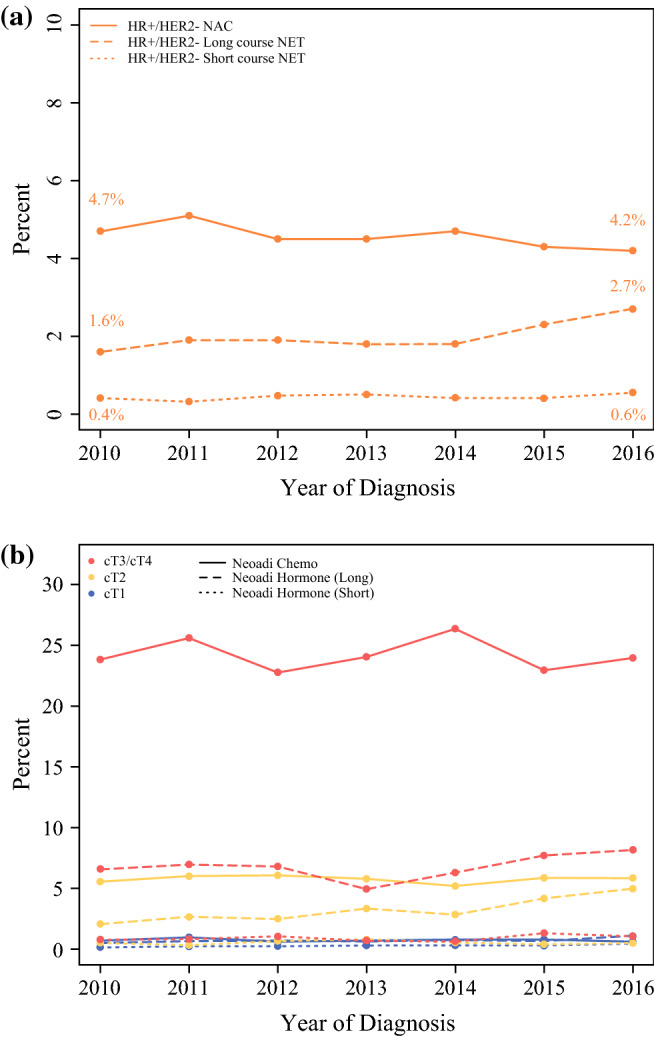
Primary treatment strategy over time in ILC cases in NCDB from 2010 to 2016. NAC use decreased while NET showed a small but significant increase over time (**a**). When stratified by T category (**b**), those with cT2 tumors had the largest increase in the use of long-course NET over time. **a** All T categories combined, **b** Treatment trends stratified by T category

Across the study period, the percentage of HR+/HER2− ILC patients treated with primary surgery did not change significantly at 93.2% in 2010, to 92.5% in 2016 (*p* = 0.20). There was a small decrease in the use of NAC (from 4.7 to 4.2%, *p* = 0.007), and a small but statistically significant increase in the use of long-course NET (1.6% to 2.7%, *p* < 0.001, Fig. 2). The use of short-course NET was very low and did not change significantly during the time period analyzed (0.4% in 2010 to 0.6% in 2016). Primary surgery remained the most common treatment modality in ILC across the time period of the study.

Examined separately by clinical T category (Fig. 2), the most notable trend was in patients with cT2 disease, where the proportion undergoing primary surgery decreased significantly from 91.8 in 2010 to 88.6% in 2016 (*p* < 0.001), driven mostly by an increase in the use of long-course NET from 2.1 in 2010 to 5.0% in 2016 (trend *p* < 0.001). In these cT2 patients, the use of NAC remained stable at 5–6% over the entire period, and the use of short-course NET was consistently < 1%. No significant changes over time were seen in the management of cT3/cT4 disease, where 68.0% underwent primary surgery, 24.2% underwent NAC, 6.8% underwent long-course NET, and 0.9% underwent short-course NET overall. In cT1 disease, 98.2% had primary surgery and 1.8% received neoadjuvant therapy overall with no large changes over time; however, very small but statistically significant changes were observed in the use of short-course NET (0.2% in 2010 to 0.5% in 2016, *p* = 0.005) and long-course NET (0.5% in 2010 to 1.1% in 2016, *p* = 0.006).

On multivariable analysis, factors associated with the choice for neoadjuvant systemic therapy instead of primary surgery included later year of diagnosis, younger age, Hispanic ethnicity, non-White race, Charlson–Deyo comorbidity score of 0, higher clinical T category (OR 5.1 cT2 versus cT1 and OR 18.6 cT3/cT4 versus cT1, each *p* < 0.001), and clinical node status (OR 2.3 for cN+ versus cN0, *p* < 0.001, Table 2).

**Table 2:** Univariate and multivariable logistic regression analyses assessing factors associated with undergoing neoadjuvant systemic treatment

Variable	Univariate odds ratio (95% CI)	*p*-value	Multivariable odds ratio (95% CI)	*p*-value
Year of diagnosis	1.01 (1.00, 1.03)	0.20	1.05 (1.03, 1.07)	< 0.001
Age group				
< 50	1.0 reference		1.0 reference	
50–59	0.74 (0.68, 0.80)	< 0.001	0.88 (0.80, 0.97)	0.007
60–69	0.60 (0.55, 0.65)	< 0.001	0.83 (0.75, 0.92)	< 0.001
70–79	0.45 (0.41, 0.49)	< 0.001	0.68 (0.60, 0.78)	< 0.001
80+	0.42 (0.37, 0.48)	< 0.001	0.55 (0.47, 0.65)	< 0.001
Spanish/Hispanic origin				
Not Spanish/Hispanic	1.0 reference		1.0 reference	
Spanish/Hispanic	1.64 (1.46, 1.85)	< 0.001	1.35 (1.18, 1.54)	< 0.001
Unknown	0.90 (0.75, 1.08)	0.25	0.86 (0.70, 1.06)	0.16
Race				
White	1.0 reference		1.0 reference	
Black	1.27 (1.16, 1.38)	< 0.001	1.21 (1.09, 1.34)	< 0.001
Other	1.27 (1.10, 1.46)	< 0.001	1.20 (1.01, 1.43)	0.04
Unknown	1.24 (0.93, 1.65)	0.14	1.50 (1.07, 2.11)	0.02
Charlson–Deyo score				
0	1.0 reference		1.0 reference	
1	0.88 (0.80, 0.96)	0.005	0.87 (0.79, 0.96)	0.005
2+	0.94 (0.79, 1.11)	0.43	0.99 (0.82, 1.19)	0.91
Primary payer				
Private insurance/managed care	1.0 reference		1.0 reference	
Medicaid	1.62 (1.44, 1.81)	< 0.001	1.26 (1.11, 1.43)	< 0.001
Medicare	0.72 (0.67, 0.76)	< 0.001	1.06 (0.96, 1.16)	0.27
Not insured	2.01 (1.66, 2.46)	< 0.001	1.46 (1.17, 1.83)	< 0.001
Other government	1.44 (1.14, 1.87)	0.003	1.40 (1.06, 1.84)	0.02
Unknown	1.28 (0.98, 1.66)	0.07	1.39 (1.05, 1.85)	0.02
Clinical T category				
cT1	1.0 reference		1.0 reference	
cT2	5.83 (5.33, 6.37)	< 0.001	5.09 (4.65, 5.58)	< 0.001
cT3/4	25.71 (23.48, 28.14)	< 0.001	18.60 (16.89, 20.48)	< 0.001
Unknown	1.97 (1.67, 2.33)	< 0.001	1.83 (1.51, 2.23)	< 0.001
Clinical node status				
cN0	1.0 reference		1.0 reference	
cN+	5.84 (5.48, 6.23)	< 0.001	2.25 (2.09, 2.42)	< 0.001
Unknown	0.92 (0.79, 1.07)	0.25	1.13 (0.93, 1.36)	0.23
Grade				
Well differentiated	1.0 reference		1.0 reference	
Moderately differentiated	1.00 (0.94, 1.08)	0.91	0.80 (0.74, 0.87)	< 0.001
Poorly differentiated/undifferentiated	1.40 (1.23, 1.58)	< 0.001	0.82 (0.72, 0.94)	0.004
Unknown	1.64 (1.48, 1.82)	< 0.001	1.33 (1.19, 1.49)	< 0.001

### Primary Treatment Strategy and Surgical Outcomes

Of the 69,312 HR+/HER2− ILC patients in the study cohort, 64,460 (93.0%) were treated with primary surgery, 3146 (4.5%) with NAC, 1401 (2.0%) with long-course NET, and 305 (0.4%) with short-course NET. Among the cases receiving NET, the median days between start of NET and surgery was 19 [interquartile range (IQR) 13–23] days for short-course NET and 132 (IQR 74–186) days for long-course NET.

**Fig. 3:**
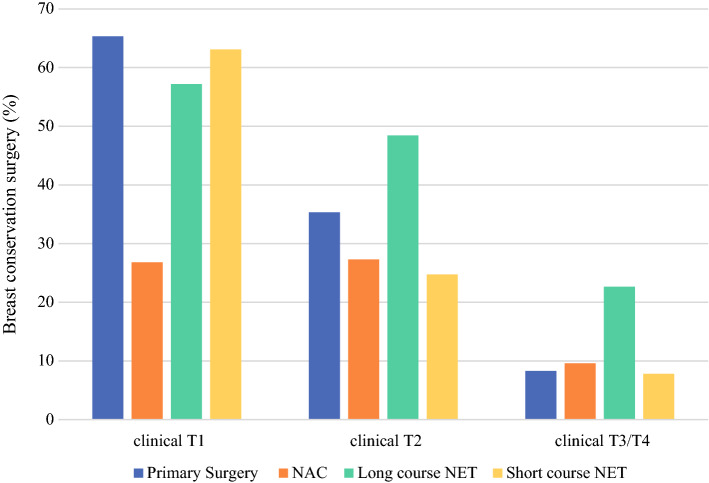
Rate of BCS by clinical T category and primary treatment strategy in HR positive/HER2 negative ILC. Long-course NET was associated with increased rates of BCS for clinical T2, T3, and T4 tumors on univariate analysis (*p* < 0.001 for all comparisons) and in a multivariable logistic regression model. NAC was associated with a small increase in BCS only in cT3/T4 tumors

Overall, 50.3% underwent BCS. BCS rates were higher with lower clinical T category (65.1% in clinical T1 cases, 35.4% in clinical T2 cases, and 9.9% in clinical T3/4 cases, *p* < 0.001). Stratified by clinical T category, primary surgery patients had the highest proportion of BCS among cT1 tumors; however, among cT2 tumors, 48.4% (295/609) of patients undergoing long-course NET underwent BCS compared with 35.3% for primary surgery, 27.3% for NAC, and 24.8% for short-course NET (*p* < 0.001). Similarly, among cT3/cT4 tumors, long-course NET was also associated with a higher BCS rate: 22.6% BCS for long-course NET, 8.3% BCS for primary surgery, 9.6% BCS for NAC, 7.8% BCS for short-course NET (*p* < 0.001, Fig. 3). Within the cohort that received long-course NET, patients undergoing BCS started their NET a median of 147 (IQR 90–202) days prior to surgery, compared with 125 (IQR 66–176) days in patients undergoing mastectomy, *p* < 0.001. Among those who received long-course NET, longer treatment duration was associated with significantly higher rates of BCS across all clinical T categories, and with lower rates of axillary dissection among clinically node-negative patients (Table 3).

**Table 3:** Rates of BCS and removal of > 5 axillary nodes stratified by duration of NET within cohort receiving long-course NET

	31–89 days (1–3 months)	90–179 days (3–6 months)	180–269 days (6–9 months)	≥ 270 days (> 9 months)	*p*-value
BCS rate					
Overall	143 (33.6%)	243 (40.9%)	128 (45.6%)	58 (59.2%)	< 0.001
cT1	78 (48.8%)	53 (65.4%)	22 (71.0%)	10 (76.9%)	0.01
cT2	56 (34.4%)	136 (48.4%)	78 (61.9%)	25 (64.1%)	< 0.001
cT3/4	6 (7.1%)	52 (23.5%)	27 (22.3%)	21 (50.0%)	< 0.001
> 5 nodes excised					
Overall	146 (36.0%)	238 (43.9%)	87 (37.2%)	25 (32.5%)	0.03
cN0	87 (26.6%)	143 (34.3%)	50 (28.6%)	9 (17.0%)	0.02
cN+	53 (77.9%)	84 (80.0%)	36 (69.2%)	13 (68.4%)	0.41

On multivariable analysis to evaluate the association between primary treatment strategy and BCS, there was a significant interaction between primary treatment strategy and clinical T category, such that treatment strategy increased the likelihood of BCS only in those with clinical T2–4 tumors but not in clinical T1 tumors (Table 4). In fact, for patients with clinical T1 tumors, those who received NAC or long-course NET were significantly less likely to undergo BCS than those who underwent primary surgery (OR 0.31, 95% CI 0.23–0.41 and OR 0.65, 95% CI 0.51–0.83, respectively). However, for those with clinical T2 tumors, long-course NET was associated with significantly higher odds for undergoing BCS (OR 1.51, 95% CI 1.28–1.79). Among the cases with clinical T3–4 tumors, both NAC and long-course NET were associated with increased odds of BCS (OR 1.47, 95% CI 1.21–1.79 and OR 2.54, 95% CI 1.98–3.24, respectively). Short-course NET was not associated with increased odds of BCS in any T category. Additional factors associated with BCS were older age, lower tumor grade, lower clinical N stage, lower Charlson–Deyo comorbidity score, and unifocal disease.

**Table 4:** Multivariable models assessing patient and treatment factors associated with BCS

Variable	Multivariable odds ratio for undergoing BCS (95% CI)	*p*-value
Year of diagnosis	1.04 (1.04, 1.05)	< 0.001
Age group (years)		
< 50	1.0 reference	
50–59	1.89 (1.79, 2.00)	< 0.001
60–69	2.70 (2.55, 2.86)	< 0.001
70–79	3.15 (2.94, 3.37)	< 0.001
80+	3.42 (3.15, 3.70)	< 0.001
Spanish/Hispanic origin		
Not Spanish/Hispanic	1.0 reference	
Spanish/Hispanic	1.01 (0.93, 1.10)	0.81
Unknown	1.02 (0.93, 1.13)	0.64
Race		
White	1.0 reference	
Black	1.33 (1.25, 1.41)	< 0.001
Other	1.04 (0.95, 1.14)	0.38
Unknown	1.53 (1.26, 1.85)	< 0.001
Charlson–Deyo score		
0	1.0 reference	
1	0.86 (0.82, 0.90)	< 0.001
2+	0.72 (0.66, 0.79)	< 0.001
Primary payer		
Private insurance/managed care	1.0 reference	
Medicaid	1.05 (0.97, 1.14)	0.22
Medicare	1.0 (0.96, 1.05)	0.85
Not insured	0.95 (0.82, 1.11)	0.53
Other government	1.0 (0.84, 1.18)	0.99
Unknown	1.22 (1.03, 1.44)	0.02
Clinical N category		
cN0	1.0 reference	
cN1	0.48 (0.45, 0.52)	< 0.001
cN2/N3	0.40 (0.35, 0.46)	< 0.001
Unknown	0.81 (0.75, 0.88)	< 0.001
Grade		
Well differentiated	1.0 reference	
Moderately differentiated	0.91 (0.87, 0.94)	< 0.001
Poorly differentiated/undifferentiated	1.0 (0.93, 1.07)	0.94
Unknown	1.08 (1.02, 1.16)	0.02
Multicentric		
No	1.0 reference	
Yes	0.36 (0.34, 0.38)	< 0.001
Primary treatment × clinical T category interaction		< 0.001*
Primary treatment effects stratified by clinical T category		
cT1 tumors only		
Long-course NET versus primary surgery	0.65 (0.51, 0.83)	
NAC versus primary surgery	0.31 (0.23, 0.41)	
Short-course NET versus primary surgery	0.84 (0.58, 1.24)	
cT2 tumors only		
Long-course NET versus primary surgery	1.51 (1.28, 1.79)	
NAC versus primary surgery	1.01 (0.88, 1.17)	
Short-course NET versus primary surgery	0.54 (0.34, 0.86)	
cT3/4 tumors only		
Long-course NET versus primary surgery	2.54 (1.98, 3.24)	
NAC versus primary surgery	1.47 (1.21, 1.79)	
Short-course NET versus primary surgery	0.82 (0.32, 2.08)	

^*^p-Value for test of interation between primary treatment type and clinical T catgory. A significant interaction means that the effect of primary treatment on undergoing BCS differed significantly across clinical T categories; thus, treatment odds ratios specific to each clinical T category were estimated

Considering the axilla, 87.4% of patients were clinically node negative whereas 12.6% were clinically node positive. Among cN0 patients with axillary surgery, 75.8% had 1–5 nodes examined, and 24.2% had > 5 nodes examined, with 1–5 nodes removed among 76.6% for primary surgery, 70.3% for long-course NET, 70.0% for short-course NET, and 55.4% for NAC (*p* < 0.001). Among cN+ patients, 19.3% had 1–5 nodes examined, and 80.7% had > 5 nodes examined overall; 23.8% of cN+ patients treated with long-course NET had 1–5 nodes removed, compared with 19.2% for primary surgery, 18.6% for NAC, and 17.0% for short-course NET. On multivariable analysis adjusting for other clinical factors, undergoing long-course NET or NAC was associated with significantly higher odds of having less extensive axillary surgery in cN+ patients (OR 1.6 and 1.4, respectively) but not cN0 patients, resulting in a significant interaction between primary treatment strategy and clinical node status (Table 5).

**Table 5:** Multivariable models assessing patient and treatment factors associated with less extensive axillary surgery (1–5 nodes removed)

Variable	Multivariable odds ratio for undergoing less extensive axillary surgery (95% CI)	*p*-value
Year of diagnosis	1.10 (1.09, 1.11)	< 0.001
Age group (years)		
< 50	1.0 reference	
50–59	1.18 (1.11, 1.25)	< 0.001
60–69	1.34 (1.26, 1.42)	< 0.001
70–79	1.48 (1.37, 1.59)	< 0.001
80+	1.56 (1.43, 1.71)	< 0.001
Spanish/Hispanic origin		
Not Spanish/Hispanic	1.0 reference	
Spanish/Hispanic	0.89 (0.82, 0.97)	0.007
Unknown	0.94 (0.85, 1.05)	0.29
Race		
White	1.0 reference	
Black	0.89 (0.84, 0.95)	< 0.001
Other	1.11 (1.0, 1.23)	0.05
Unknown	1.33 (1.07, 1.64)	0.009
Charlson–Deyo score		
0	1.0 reference	
1	0.87 (0.82, 0.91)	< 0.001
2+	0.73 (0.66, 0.81)	< 0.001
Primary payer		
Private insurance/managed care	1.0 reference	
Medicaid	0.89 (0.82, 0.97)	0.006
Medicare	0.99 (0.94, 1.04)	0.69
Not insured	0.89 (0.76, 1.04)	0.15
Other government	0.99 (0.83, 1.19)	0.92
Unknown	1.05 (0.87, 1.25)	0.62
Clinical T category		
cT1	1.0 reference	
cT2	0.48 (0.46, 0.50)	< 0.001
cT3/4	0.26 (0.24, 0.27)	< 0.001
Unknown	0.69 (0.63, 0.75)	< 0.001
Grade		
Well differentiated	1.0 reference	
Moderately differentiated	0.82 (0.78, 0.85)	< 0.001
Poorly differentiated/undifferentiated	0.69 (0.63, 0.74)	< 0.001
Unknown	0.86 (0.80, 0.92)	< 0.001
Multicentric		
No	1.0 reference	
Yes	0.75 (0.71, 0.79)	< 0.001
Primary treatment × clinical T category interaction		< 0.001*
Primary treatment effects stratified by clinical node status		
cN0 tumors only		
Long-course NET versus primary surgery	1.09 (0.94, 1.26)	
NAC versus primary surgery	0.84 (0.74, 0.94)	
Short-course NET versus primary surgery	0.87 (0.64, 1.17)	
cN+ tumors only		
Long-course NET versus primary surgery	1.56 (1.15, 2.14)	
NAC versus primary surgery	1.37 (1.18, 1.58)	
Short-course NET versus primary surgery	0.96 (0.43, 2.11)	

^*^p-Value for test of interation between primary treatment type and clinical node status. A significant interaction means that the effect of primary treatment on undergoing less extensive axillary surgery differed significantly by clinical N status; thus, treatment odds ratios specific to each clinical node category were estimated

### HER2+ and TNBC Subsets

There were 4651 patients (3668 HER+ and 983 TNBC) with biologic subtype other than HR+/HER2−, representing 6.0% of all stage I–III ILC cases in this database. Of these, 80.1% were treated with primary surgery and 19.1% with NAC; there were also a small number of patients with HR+/HER2+ disease who were treated with long-course (0.7%) or short-course (0.2%) NET. In those with HER2-overexpressing or triple-negative tumors, the number of patients receiving NAC increased significantly between 2010 and 2016 (from 12.2 to 29.1%, and from 12.1 to 23.7%, respectively, *p* < 0.01 for both).

## Discussion

In this analysis of nearly 70,000 HR+/HER2− ILC cases from the NCDB, we found small but significant changes in primary treatment strategy over time, with a decrease in the use of NAC and an increase in long-course NET. For larger tumors, neoadjuvant approaches were associated with increased rates of BCS. In particular, among the clinical T2 cases, long-course NET use was associated with increased odds of BCS. Among the clinical T3/4 tumors, both long-course NET and NAC use resulted in higher odds of BCS. Interestingly, clinical T1 tumors were more likely to be treated with BCS if the primary treatment strategy was surgery. For those clinical T1 tumors receiving NAC or NET, BCS was significantly less likely. This suggests that these neoadjuvant strategies were employed for reasons other than downstaging tumors to achieve BCS, which is consistent with small tumor sizes at baseline in this group. Potential reasons for neoadjuvant therapy in this group include attempting to evaluate response to therapy, or a desire to delay surgical intervention.

While other data show that use of NAC has increased for all breast cancer subtypes, national trends show the largest increase in the triple-negative and HER2+ subtypes, likely reflecting selection of patients who are most likely to have robust response.^20^,^21^ We found this to be true in ILC as well, despite the very small proportion with triple-negative and HER2+ disease. These results are consistent with publications showing that, although ILC tumors overall have low response rates to NAC, the subset of ILC with high-risk biology appears to garner similar benefit as triple-negative or HER2+ invasive ductal carcinoma.^22^,^23^ This represents an appropriate tailoring of therapy not only by histologic subtype, but by receptor subsets within ILC.

Our analysis also found that long-course NET was associated with significantly higher rates of BCS in ILC tumors that were clinical T2 or greater, which is consistent with prior literature.^24^ Although NET is associated with increased rates of BCS, the lack of survival improvement and some data showing that primary surgery with adjuvant therapy may be associated with better survival has led some investigators to question the value of neoadjuvant approaches in ILC altogether.^2^,^9^,^10^ Combined with current data highlighting the potential benefit of short-course NET for tailoring subsequent treatment, we wondered whether this approach had become more common in ILC. We found no such increase but hypothesize that, until further data emerge supporting the use of change in Ki67 to determine management, this approach may remain confined to surgical window trials.

We found an association between neoadjuvant therapy and extent of nodal surgery in clinically node-positive patients. Those who received long-course NET or NAC had significantly higher odds of undergoing less extensive axillary surgery (1–5 nodes removed versus > 5). These findings differ from a prior analysis of node-positive ILC cases in which NAC was not found to be associated with number of nodes removed.^9^ This difference could be explained by our choice to evaluate number of nodes removed as a categorical variable representing typical number of nodes removed in sentinel lymph node surgery versus axillary dissection. This suggests that neoadjuvant approaches can downstage the axilla in ILC and potentially allow for omission of axillary dissection if the type and duration of neoadjuvant therapy is tailored to tumor type. However, the absence of axillary dissection does not imply nodal pathological complete response, and data confirm a decrease in performance of axillary dissection even among patients without complete nodal response to neoadjuvant therapy.^25^ Since providers who utilize neoadjuvant therapy at higher rates may also be less likely to recommend axillary dissection for residual nodal disease, this is an important potential confounder to consider in retrospective analyses of neoadjuvant therapy and surgical outcomes.

Overall, these findings are consistent with the increased appreciation for tumor biology in ILC and potential for increased responsiveness to endocrine therapy instead of chemotherapy. Recent analyses have identified molecular subtypes that are specific to ILC, and separate ILC into subtypes that may have differing responses to therapy.^26^–^28^ This subtyping at the molecular level may allow for further tailoring of therapy in ILC.

While this study includes a large number of pure ILC cases, the retrospective nature of this analysis limits the ability to draw conclusions about why particular treatment strategies were chosen; For example, short-course NET may have been used to evaluate change in Ki67, but these data are not available in the NCDB. Furthermore, accurate clinical staging in ILC is difficult due to lower sensitivity of imaging tests, which could impact analyses by stage.

These findings suggest that appropriate selection of ILC patients for neoadjuvant approaches can improve surgical outcomes. Additionally, these findings support the use of longer courses of NET when utilized in ILC. The low rates at which neoadjuvant approaches are currently used in this strongly HR+/HER2− tumor type suggest room for potential improvement in care. Further work in identifying predictors of response to therapy, and development of imaging tools to accurately monitor response are needed for patients with ILC.
